# Excessively low salt diet damages the heart through activation of cardiac (pro) renin receptor, renin-angiotensin-aldosterone, and sympatho-adrenal systems in spontaneously hypertensive rats

**DOI:** 10.1371/journal.pone.0189099

**Published:** 2017-12-08

**Authors:** Chihiro Okamoto, Yuka Hayakawa, Takuma Aoyama, Hisaaki Komaki, Shingo Minatoguchi, Masamitsu Iwasa, Yoshihisa Yamada, Hiromitsu Kanamori, Masanori Kawasaki, Kazuhiko Nishigaki, Atsushi Mikami, Shinya Minatoguchi

**Affiliations:** Department of Cardiology, Gifu University Graduate School of Medicine, Yanagido, Gifu, Japan; Max Delbruck Centrum fur Molekulare Medizin Berlin Buch, GERMANY

## Abstract

**Objective:**

A high salt intake causes hypertension and leads to cardiovascular disease. Therefore, a low salt diet is now recommended to prevent hypertension and cardiovascular disease. However, it is still unknown whether an excessively low salt diet is beneficial or harmful for the heart.

**Methods:**

Wistar Kyoto rats (WKYs) and spontaneously hypertensive rats (SHRs) received normal salt chow (0.9% salt diet) and excessively low salt chow (0.01% salt diet referred to as saltless diet) for 8 weeks from 8 to 16 weeks of age. The effects of the excessively low salt diet on the cardiac (pro) renin receptor, renin-angiotensin-aldosterone, and sympatho-adrenal systems were investigated.

**Results:**

The excessively low salt diet did not affect the systolic blood pressure but significantly increased the heart rate both in WKYs and SHRs. The excessively low salt diet significantly elevated plasma renin activity, plasma angiotensin I, II and aldosterone concentrations, and plasma noradrenaline and adrenaline concentrations both in WKYs and SHRs. Cardiac expressions of renin, prorenin, (P)RR, angiotensinogen, and angiotensin II AT1 receptor and phosphorylated (p)-ERK1/2, p-HSP27, p-38MAPK, and TGF-ß1 were significantly enhanced by the excessively low salt diet in both WKYs and SHRs. The excessively low salt diet accelerated cardiac interstitial and perivascular fibrosis and increased the cardiomyocyte size and interventricular septum thickness in WKYs and SHRs but the extent was greater in SHRs.

**Conclusion:**

An excessively low salt diet damages the heart through activation of plasma renin-angiotensin-aldosterone and sympatho-adrenal systems and activation of cardiac (P)RR and angiotensin II AT1 receptor and their downstream signals both in WKYs and SHRs.

## Introduction

It has been reported that excessive salt intake increases the blood pressure and the restriction of salt intake decreases blood pressure [1]. A high salt intake has been reported to increase the risk of cardiovascular disease [2]. We previously reported that a high salt intake enhanced cardiac expressions of prorenin, renin, and (pro)renin receptor [(P)RR] as well as cardiac angiotensinogen and angiotensin II AT1 receptor, and activated their downstream signals ERK1/2, TGF-β1, p38MAPK, and HSP27, leading to the acceleration of cardiac interstitial fibrosis, perivascular fibrosis, and cardiomyocyte hypertrophy and to the deterioration of the cardiac function in spontaneously hypertensive rats (SHRs) [3]. It is recommended that salt intake should be less than 6 g/day in patients with hypertension [4]. Restriction of salt intake has been reported to decrease blood pressure and reduce mortality and morbidity due to cardiovascular disease [5, 6]. On the other hand, it has recently been reported that large-scale prospective cohort clinical trials demonstrated that subjects with a low salt diet were associated with higher rates of cardiovascular disease morbidity and mortality compared with those with a normal or high salt diet [7, 8]. At first glance, these results are confusing because they are different from the common sense view that a high salt intake is associated with an increased risk of cardiovascular disease [2]. However, they may be due to activation of the plasma renin-angiotensin-aldosterone system or sympatho-adrenal system, which is caused by an excessively low salt diet. In fact, a higher plasma renin activity has been reported to be associated with an increased risk of myocardial infarction [9], as well as higher cardiac morbidity and mortality in patients with coronary artery disease [10]. Furthermore, cardiac patients with higher plasma norepinephrine levels showed a poor prognosis and shortened life expectancy [11]. It has also been reported that a low salt diet was associated with the sympathetic nervous system in patients with hypertension [12].

Generally, plasma renin activity increases with a decrease in salt intake and decreases with an increase in salt intake [13, 14]. However, it is still unclear whether the cardiac tissue renin angiotensin system (RAS) including classical RAS such as angiotensinogen, angiotensin II, and angiotensin II AT1 receptor, and the recently identified prorenin-(P)RR system and its downstream signals, are activated by an excessively low salt diet. Therefore, in the present study, we investigated the effect of an excessively low salt diet on plasma renin-angiotensin-aldosterone, sympatho-adrenal, cardiac tissue renin angiotensin, and prorenin-(P)RR systems, their signal transduction, and the development of cardiac damage and function in WKYs and SHRs.

## Materials and methods

### Experimental animals

Male 8-week-old spontaneously hypertensive rats (SHRs) and Wistar Kyoto rats (WKYs), purchased from Chubu Kagaku Sizai (Nagoya, Japan), were maintained in a temperature and humidity controlled animal room with a 12-h light and 12-h dark cycle. All rats were handled in accordance with the Guide for the Care and Use of Laboratory Animals, published by the US National Institutes of Health (NIH publication 85–23, revised in 1996). The study protocol was approved by the Committee for Animal Research and Welfare of Gifu University, Gifu, Japan (Permit Number 29–20). At the end of the experiment, all rats were anesthetized with pentobarbital sodium (30~40 mg/kg, i.p.) and a catheter was cannulated into the femoral vein to take blood samples, and then the rats were sacrificed by an overdose of pentobarbital. All efforts were made to minimize pain and suffering.

### Protocol

Spontaneously hypertensive rats (SHR) and Wistar Kyoto rats (WKY) at 8 weeks old received regular rat chow (0.9% NaCl diet, CE-2; CLEA Japan, Inc., Tokyo, Japan) and excessively low salt rat chow (0.01% NaCl diet, 0.01% NaCl chou was prepared from AIN-93G, Oriental Yeast Co. Ltd., Tokyo, Japan) for 8 weeks from 8 to 16 weeks of age (n = 10, respectively). We regarded a diet containing 0.9%NaCl as a normal diet for rats and defined as a normal diet in the present study. We defined a diet containing 0.01% NaCl as an excessively low salt diet.

The study groups were WKY+normal salt (WKY+NS), WKY+excessively low salt (WKY+ELS), SHR+normal salt (SHR+NS), and SHR+excessively low salt (SHR+ELS) groups.

### Measurement of blood pressure and heart rate

Systolic blood pressure and heart rate were measured once a week for 8 weeks by the tail-cuff method in all rats.

### Echocardiography

Echocardiography (Vevo 770; Visualsonics, Toronto, Canada, equipped with a 45-MHz imaging transducer) was performed at 8 and 16 weeks of age, and LV fractional shortening (FS) and the interventricular septum thickness (IVSth) were obtained.

### Plasma renin activity and angiotensin I, II and aldosterone concentrations

At the end of the experiment, all rats were anesthetized with pentobarbital sodium (30~40 mg/kg, i.p.) and a catheter was cannulated into the carotid artery to take blood samples (~8 ml each) for the measurement of plasma renin activity and plasma angiotensin I, II and aldosterone concentrations. Plasma renin activity and plasma angiotensin I, II and aldosterone concentrations were measured using the RIA2 method (SRL, Tokyo, Japan).

### Plasma noradrenaline and adrenaline concentrations

From the blood taken at the end of the experiment, plasma noradrenaline and adrenaline concentrations were measured using HPLC coupled with electrochemical detection (SRL, Tokyo, Japan).

### Western blot analysis

At 16 weeks of age, the animals were sacrificed by an overdose of anesthesia and the heart was excised. Western blot analysis was performed using lysates from the heart tissues. Proteins were separated and transferred to membranes using standard protocols. Then, they were probed with antibodies against prorenin and renin (1:1000, Santa Cruz Biotechnology, Inc.), (P)RR (1:1000, Abcam), angiotensinogen (1:200, Phoenix Pharmaceuticals, Inc.), angiotensin II AT1 receptor (1:200, Enzo Life Science), extracellular signal-related kinases (ERK)1/2 (1:1000, Cell Signaling Technology, Inc.), phosphorylated (p)-ERK1/2 (1:100; Cell Signaling Technology, Inc.), transforming growth factor (TGF)-ß1(1:2000; Santa Cruz Biotechnology, Inc.), p38 mitogen-activated protein kinase (p38MAPK) (1:1000; Cell Signaling Technology, Inc.), p-p38MAPK (1:1000; Cell Signaling Technology, Inc.), heat shock protein (HSP)27 (1:1000; Santa Cruz Biotechnology, Inc.), and p-HSP27 (1:1000; Santa Cruz Biotechnology, Inc.). The blots were visualized by chemiluminescence (ECL Prime, Amersham), and the signals were quantified by densitometry. α-Tubulin (1:1000, Cell Signaling Technology, Inc.) served as the loading control.

### Pathology

At 16 weeks of age at the end of the experiment, the animals were sacrificed by an overdose of anesthesia and the heart was excised and the LV was then weighed and sectioned into three transverse slices parallel to the atrioventricular ring. The slices were then fixed in 10% buffered formalin for 4 hours, embedded in paraffin, and then cut into 4-μm-thick sections with a microtome. These sections were stained with hematoxylin-eosin and Masson’s trichrome. The preparations were observed by light microscopy and pathological findings were obtained by two persons blinded to treatment.

### Statistical analysis

All values are presented as means ± SEM. Differences among the groups were assessed by ANOVA combined with Bonferroni’s post-hoc test for multiple comparisons. Values of p < 0.05 were considered significant.

## Results

### Blood pressure and heart rate

**Fig 1:**
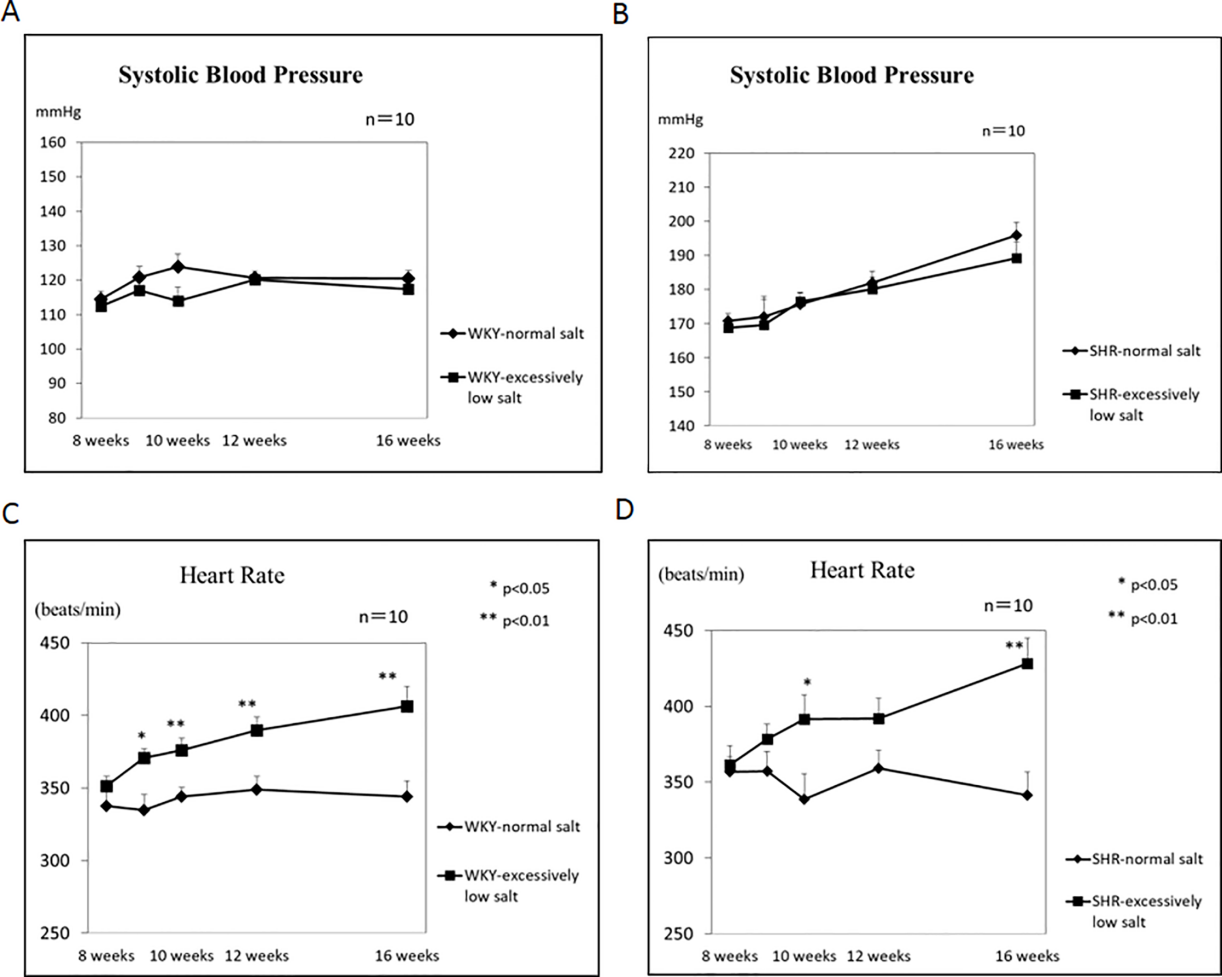
Time course changes in the systolic blood pressure and heart rate in response to the excessively low salt diet in the WKY and SHR groups.

There was no significant difference in the systolic blood pressure at any time point from 8 to 16 weeks of age between the WKY+NS and WKY+ELS groups (Fig 1A). The systolic blood pressure gradually increased from 8 to 16 weeks of age both in the SHR+NS and SHR+SL groups. However, there was no significant difference in the systolic blood pressure at any point from 8 to 16 weeks of age between the SHR+NS and SHR+ELS groups (Fig 1B). In contrast, the heart rate was significantly higher in the WKY+ELS group than in the WKY+NS group (Fig 1C) and was significantly higher in the SHR+ELS group than in the SHR+NS group (Fig 1D).

### Heart weight, weight/body weight and body weight

**Fig 2:**
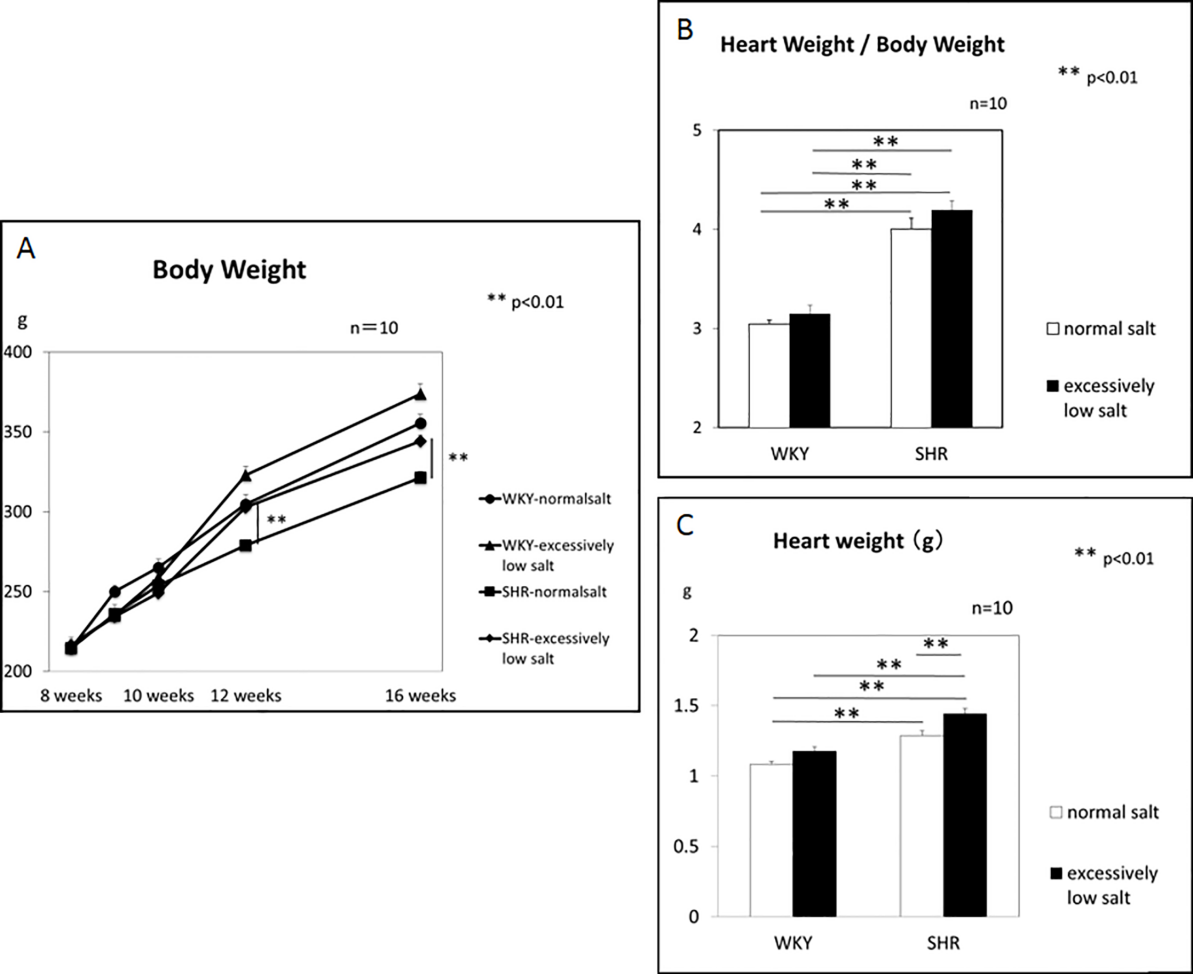
Changes in the body weight, heart weight, and heart weight/body weight ratio.

The body weight increased with time, and there was a significant difference between the SHR+NS and SHR+ELS groups at 12 and 16 weeks of age (Fig 2A). At 16 weeks of age, the heart weight/body weight was significantly greater in the SHR+NS and SHR+ELS groups than in the WKY+NS and WKY+SL groups, respectively (Fig 2B), but it was not different between the SHR+NS and SHR+ELS groups or between the WKY+NS and WKY+ELS groups, respectively, although the heart weight/body weight in the SHR+ELS group was significantly higher than that in the SHR+NS group (Fig 2B). The heart weight was significantly greater in the SHR+NS and SHR+ELS groups than in the WKY+NS and WKY+ELS groups, respectively (Fig 2C). The heart weight was significantly greater in the SHR+ELS group than in the SHR+NS group, but there was no significant difference between WKY+NS and WKY+ELS groups (Fig 2C).

### Echocardiography

**Fig 3:**
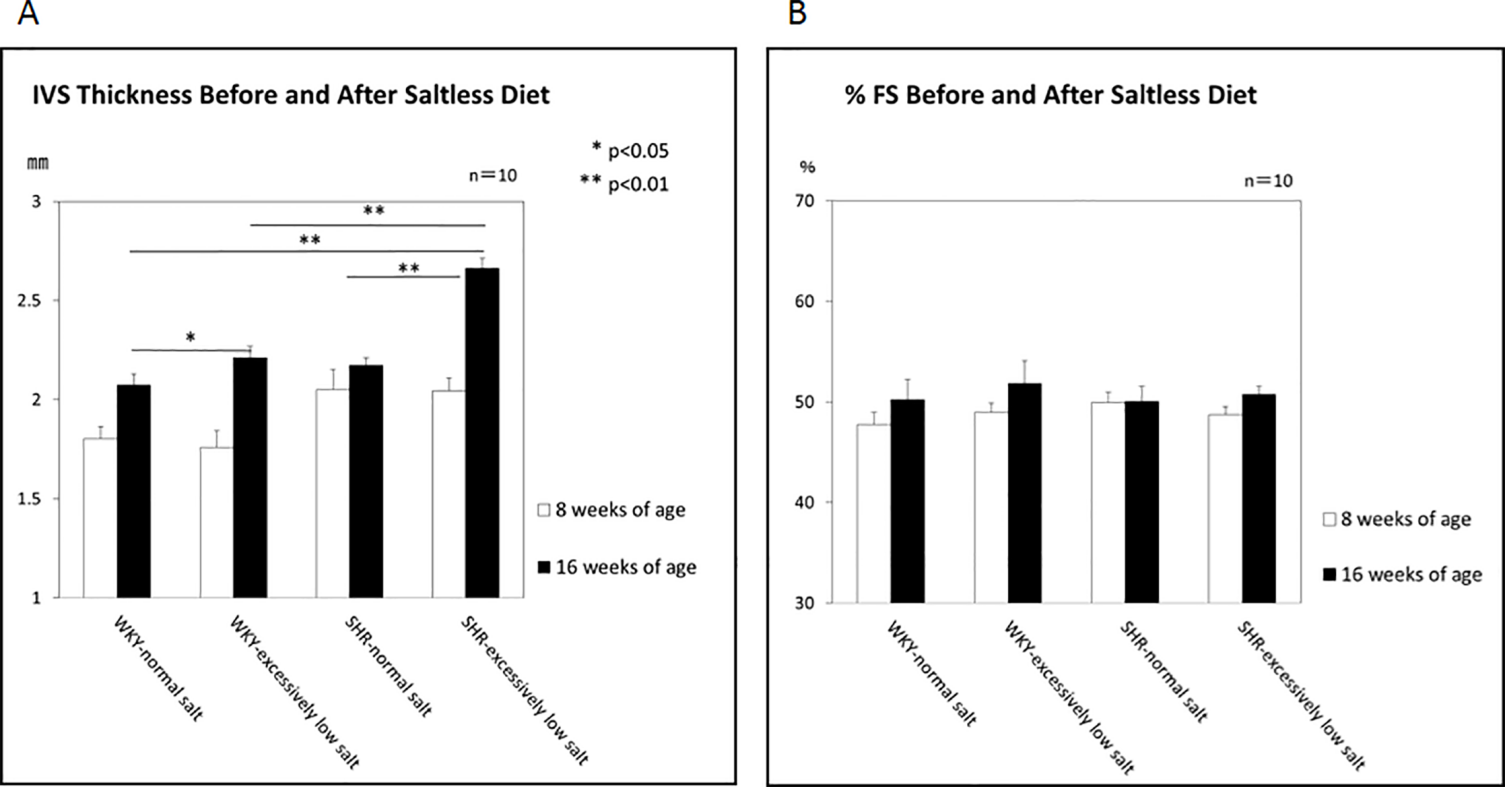
Echocardiographic findings. A: Interventricular septum (IVS) thickness at 8 and 16 weeks of age. B: LV fractional shortening (FS) at 8 and 16 weeks of age.

The interventricular septum thickness (IVSth), an indicator of left ventricular (LV) hypertrophy, was significantly greater in the WKY+ELS group than in the WKY+NS group, and in the SHR+ELS group than in the SHR+NS group (Fig 3A). There was no significant difference in LV fractional shortening (FS) among the 4 groups (Fig 3B).

### Plasma renin-angiotensin-aldosterone system

**Fig 4:**
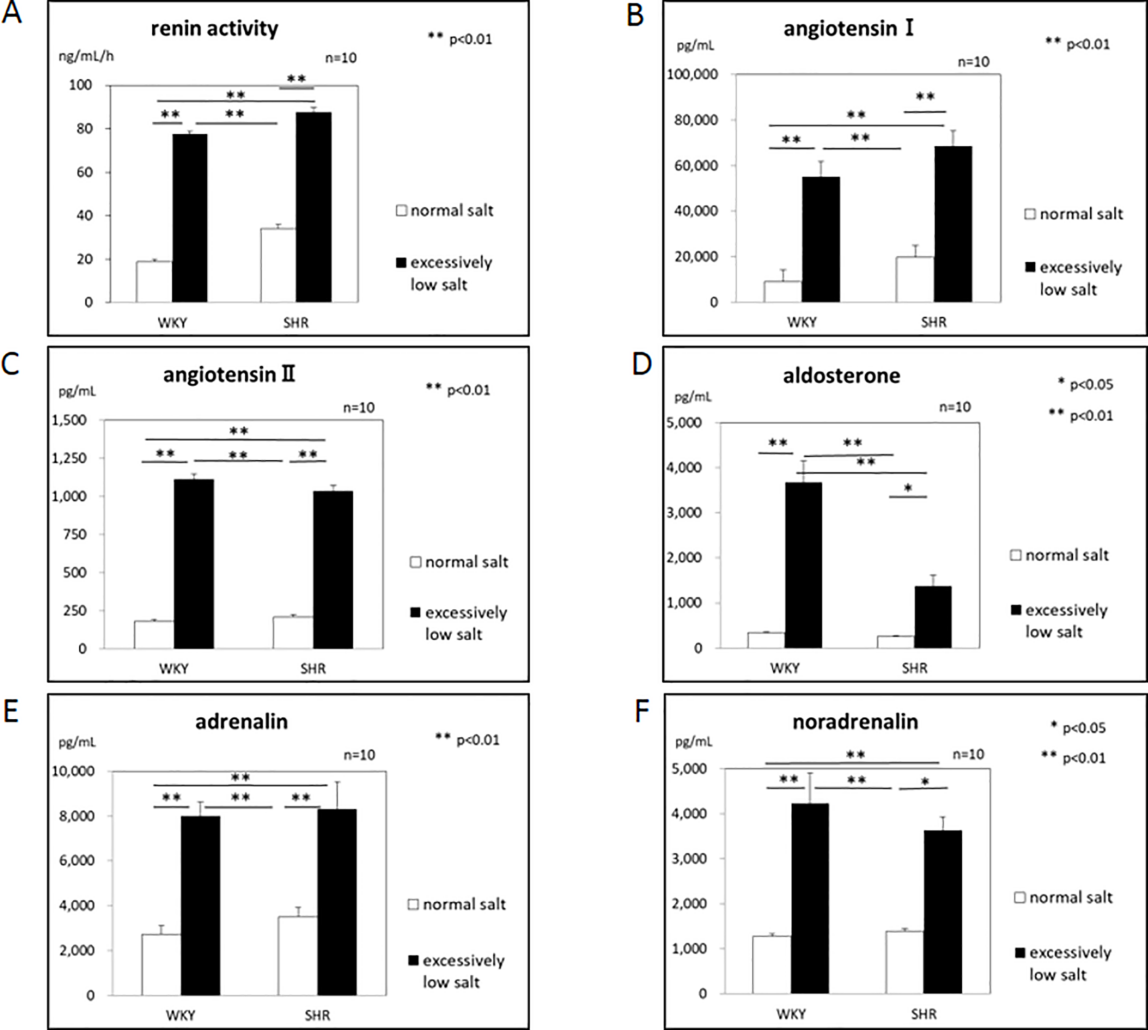
A: plasma renin activity. B: plasma angiotensin I concentration. C: plasma angiotensin II concentration. D: plasma aldosterone concentration. E: plasma noradrenaline concentration. F: plasma adrenaline concentration.

The excessively low salt diet significantly increased plasma renin activity (PRA), the plasma angiotensin I and II concentrations, and plasma aldosterone concentration both in WKYs and SHRs as compared with the normal salt diet (Fig 4A, 4B, 4C and 4D).

### Plasma noradrenaline and adrenaline concentrations

The excessively low salt diet significantly increased plasma noradrenaline and adrenaline concentrations both in WKYs and SHRs as compared with the normal salt diet (Fig 4E and 4F).

### Expression of cardiac tissue renin and (P)RR

**Fig 5:**
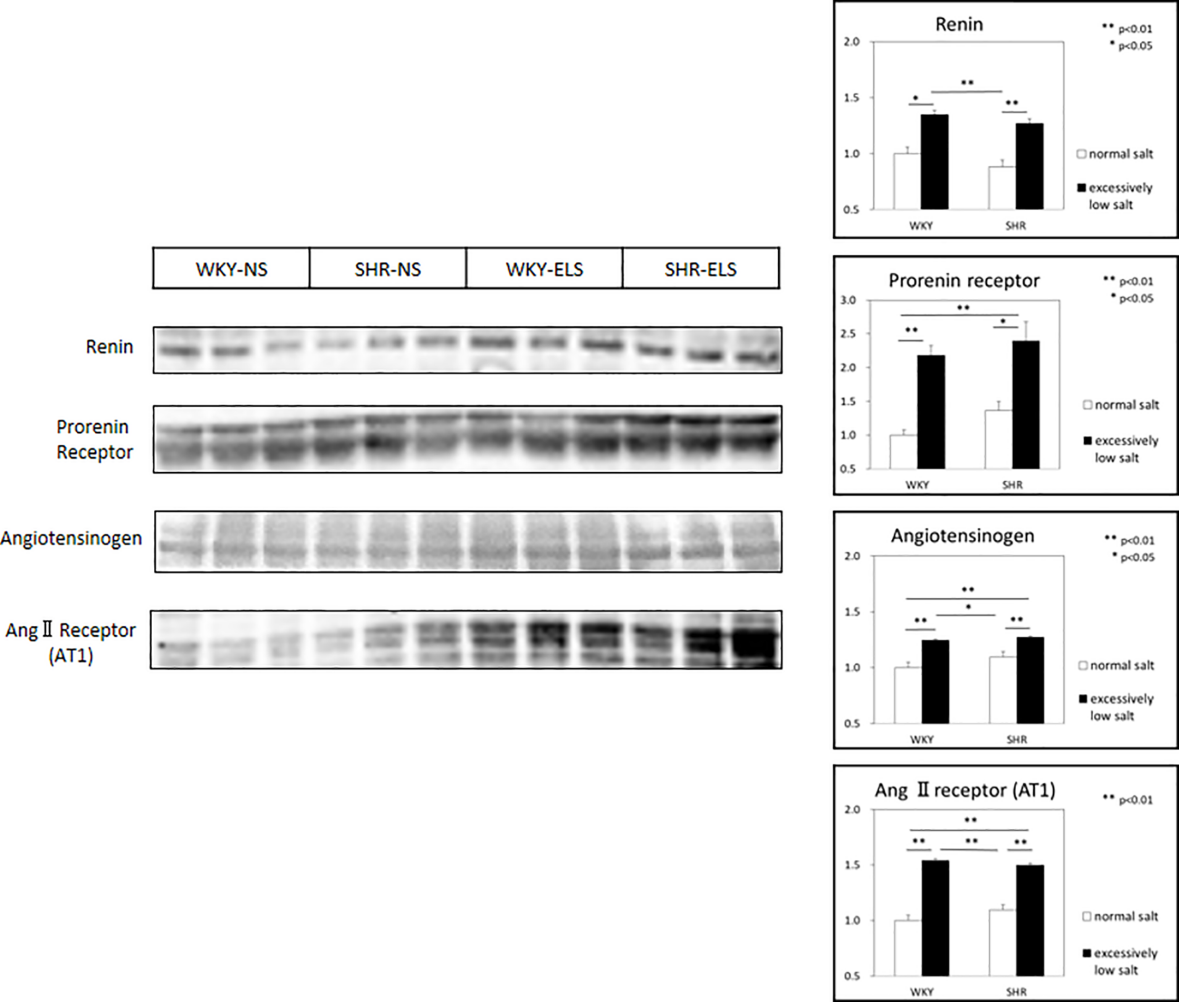
Expression of cardiac tissue renin, (pro) renin receptor, and angiotensinogen and angiotensin II AT1 receptor. Normal salt = NS, Excessively low salt = ELS, *: p<0.05, **: p<0.01.

Western blot analysis demonstrated that cardiac tissue expressions of renin and (P)RR were significantly higher in the WKY+ELS group than in the WKY+NS group, and significantly higher in the SHR+ELS group than in the SHR+NS group (Fig 5).

### Expression of cardiac tissue angiotensinogen and angiotensin II AT1 receptors

Cardiac tissue expressions of angiotensinogen and angiotensin II AT1 receptor were significantly higher in the SHR+ELS group than in the SHR+NS group, and significantly higher in the WKY+ELS group than in the WKY+NS group (Fig 5).

### Signal transduction

**Fig 6:**
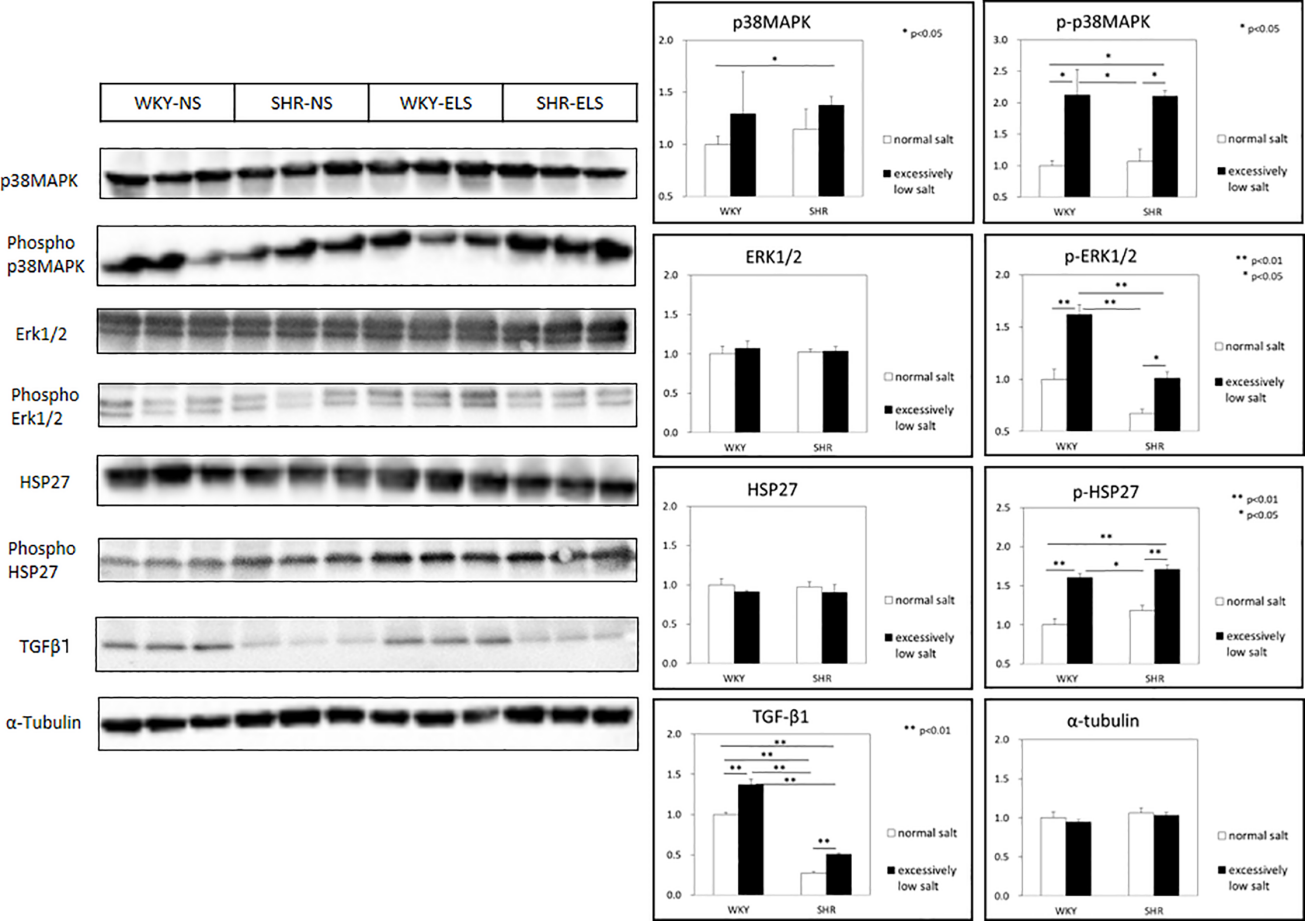
Expression of cardiac tissue ERK1/2, phospho-ERK1/2, p38MAPK, phospho-p38MAPK, HSP27, phospho-HSP27,TGF-ß1. Normal salt = NS, Excessively low salt = ELS, *: p<0.05, **: p<0.01.

Cardiac expressions of phosphorylated (p)-ERK1/2, p-p38MAPK, p-HSP27, and TGF-ß1 were significantly higher in the SHR+ELS group than in the SHR+NS group, and significantly higher in the WKY+ELS group than in the WKY+NS group, respectively (Fig 6).

### Pathology

**Fig 7:**
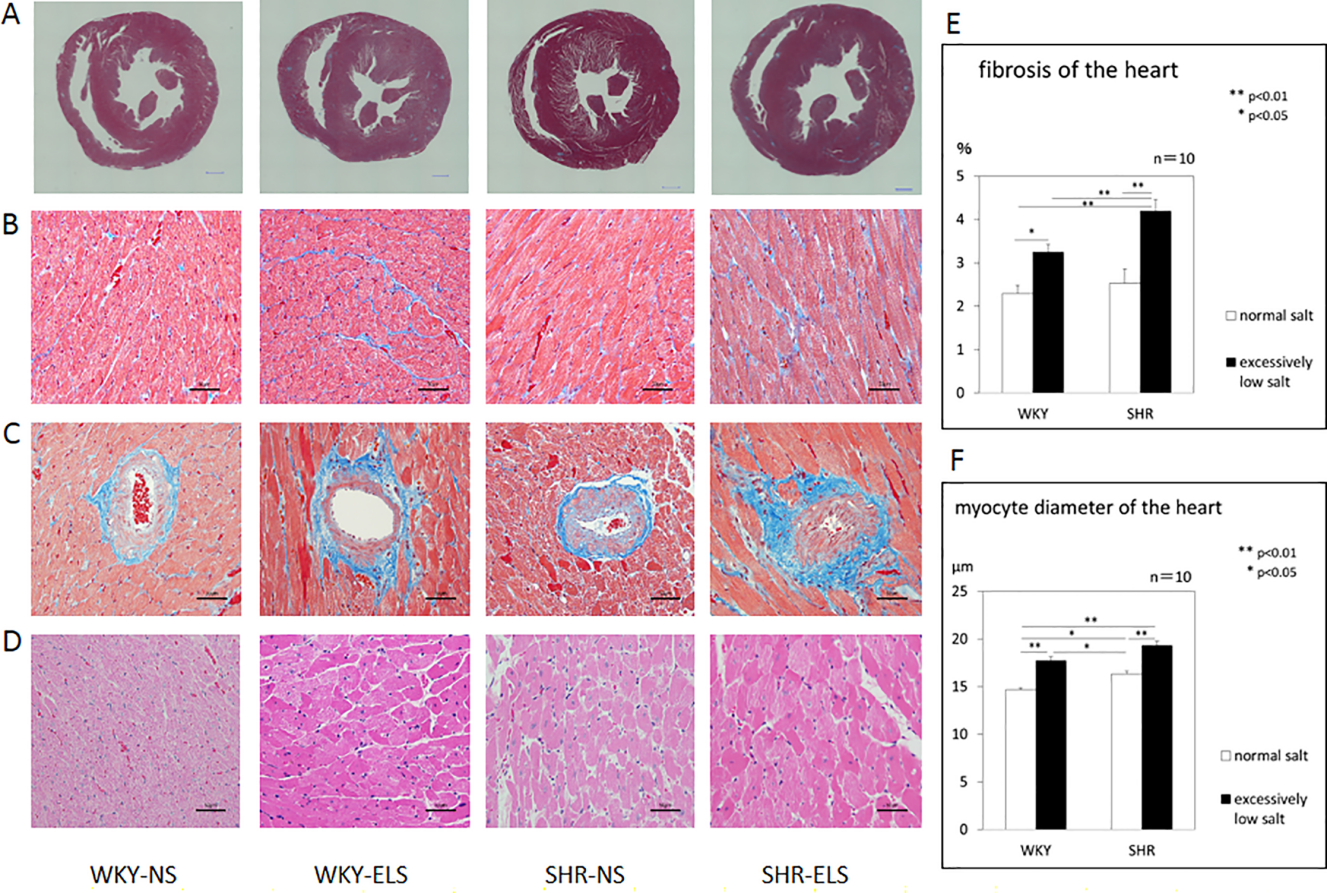
Pathology of left ventricle. A: Representative short-axis sections of cardiac ventricle stained with Masson-trichrome. Scale bar: 1000 μm. B: Representative short-axis images of myocardium and intramuscular arteries with perivascular and interstitial fibrosis stained with Masson-trichrome. Scale bar: 50 μm. C: Representative short-axis images of myocardium and interstitial fibrosis stained with Masson-trichrome. Scale bar: 50 μm. D: Representative short-axis images of the myocardium stained with hematoxylin-eosin. Scale bar: 50 μm. E: Ratio of fibrosis area/myocardium. F: Mean diameter of cardiomyocytes at 16 weeks of age. Normal salt = NS, Excessively low salt = ELS, *: p<0.05, **: p<0.01.

Fig 7A shows representative short axis sections of a left ventricle stained with Masson’s-trichrome. The excessively low salt diet accelerated the development of myocardial interstitial fibrosis and perivascular fibrosis both in SHRs and WKYs (Fig 7B and 7C). Fig 7E shows the ratio of the myocardial interstitial fibrosis area /myocardium. The ratio of myocardial interstitial fibrosis /myocardium was significantly higher in the SHR+ELS group than in the SHR+NS group, and was significantly higher in the WKY+ELS group than in the WKY+NS group. Fig 7D shows representative short-axis sections of a left ventricle stained with hematoxylin-eosin. Fig 7F shows the mean diameter of cardiomyocytes. The diameter of cardiomyocytes was significantly larger in the SHR+ELS group than in the SHR+NS group, and significantly larger in the WKY+ELS group than in the WKY+NS group.

## Discussion

### Blood pressure and heart rate

In the present study, time course changes in systolic blood pressure did not differ between the normal salt-diet and extensively low salt-diet both in the WKY and SHR groups, as shown in Fig 1A and 1B. It was reported that an excessively low salt diet prevented the development of hypertension in SHRs [15]. Our results may differ from those of this report because we used an excessively low salt diet. On the other hand, the excessively low salt diet significantly increased the time course changes in the heart rate as compared with those of the normal diet both in the WKY and SHR groups (Fig 1C and 1D). Then, why did the excessively low salt diet not decrease the systolic blood pressure but increase heart rate? One possible explanation for these results may be the activation of the renin-angiotensin and sympatho-adrenal systems. In the present study, plasma renin activity (PRA), plasma angiotensin I and II, plasma aldosterone, and plasma noradrenaline and adrenaline concentrations were significantly higher in the excessively low salt diet group than in the normal salt diet group in both the WKY and SHR groups (Fig 4A–4F). A high plasma angiotensin II concentration increases the systolic blood pressure by stimulating angiotensin II AT1 receptors [16], and a high plasma aldosterone concentration is related to hypertension [17]. Activation of the sympathetic nervous system increases the systolic blood pressure by stimulating α-adrenergic receptors in the systemic arteries [18]. Activation of the sympatho-adrenal system increases the heart rate by stimulating ß-receptors in the heart [19]. Therefore, although an excessively low salt diet might have the ability to decrease the systolic blood pressure, activation of the renin-angiotensin-aldosterone and sympatho-adrenal systems might have canceled out a decrease in the systolic blood pressure brought about by an excessively low salt diet, and so the systolic blood pressure did not decrease with an excessively low salt diet in the present study.

The excessively low salt diet significantly increased plasma noradrenaline and adrenaline concentrations both in SHRs and WKYs, suggesting the activation of the sympatho-adrenal system. The reason why an excessively low salt diet promotes activation of the sympathetic nervous system remains to be fully clarified. However, it was reported that the renal sympathetic nerve activity of rats was enhanced [20] and renal angiotensin I and II contents were enhanced in rats administered a low salt diet as compared with those receiving a normal salt diet [20]. Plasma renin activity (PRA) and plasma angiotensin II were higher in rats with a low salt diet compared with those receiving a normal salt diet [20]. These data are consistent with those in the present study, although our data focused on the heart.

### Cardiac tissue (P)RR, angiotensin II AT1 receptor, and signal transduction

In the present study, the expressions of prorenin, renin, and (pro)renin receptor in the left ventricle were significantly enhanced in the SHR group (Fig 5). These suggest that the cardiac tissue renin-prorenin-(P)RR system is activated in the SHR group. Prorenin and renin bound to (pro)renin receptor have been reported to trigger intracellular signaling and activate extracellular signal-related kinases (ERK)1/2 [21, 22], leading to the upregulation of TGF-ß and eventually fibrosis [23, 24]. It has been reported that the activation of (P)RR contributes to the development of cardiac fibrosis in SHRs [3, 25]. In the present study, we observed the activation of ERK1/2 and upregulation of TGF-ß (Fig 6) in cardiac tissues, leading to cardiac interstitial fibrosis and perivascular fibrosis in the SHR+ELS group.

The excessively low salt diet significantly enhanced myocardial expression of angiotensin II AT1 receptor in SHRs but not in WKYs (Fig 5). It was recently reported that the fibrotic response to angiotensin II is mediated by the angiotensin II AT1 receptor and requires p38MAPK phosphorylation and the subsequent increase in the expression of TGF-ß1 but does not require ERK 1/2 phosphorylation in skeletal muscle cells [26]. Therefore, downstream signals of angiotensin II AT1 receptor are p38MAPK and TGF-ß. In the present study, the phosphorylation of p38MAPK was enhanced and TGF-ß was activated in the SHR+ELS group. These results suggest that the excessively low salt diet enhanced the myocardial expression of angiotensinogen and angiotensin II AT1 receptor, and activation of p38MAPK and TGF-ß.

### Pathology

Pathologically, in the present study, cardiac interstitial fibrosis and perivascular fibrosis and cardiomyocyte hypertrophy were more frequently observed in both the WKY+ELS and SHR+ELS groups as compared with the WKY+NS and SHR+NS groups, respectively (Fig 7), suggesting that an excessively low salt diet accelerates cardiac interstitial fibrosis and perivascular fibrosis and cardiomyocyte hypertrophy through activation of (P)RR and angiotensin II AT1 receptor and subsequent activation of ERK1/2 and TGF-ß, and activation of p38 MAP kinase and HSP27.

**Fig 8:**
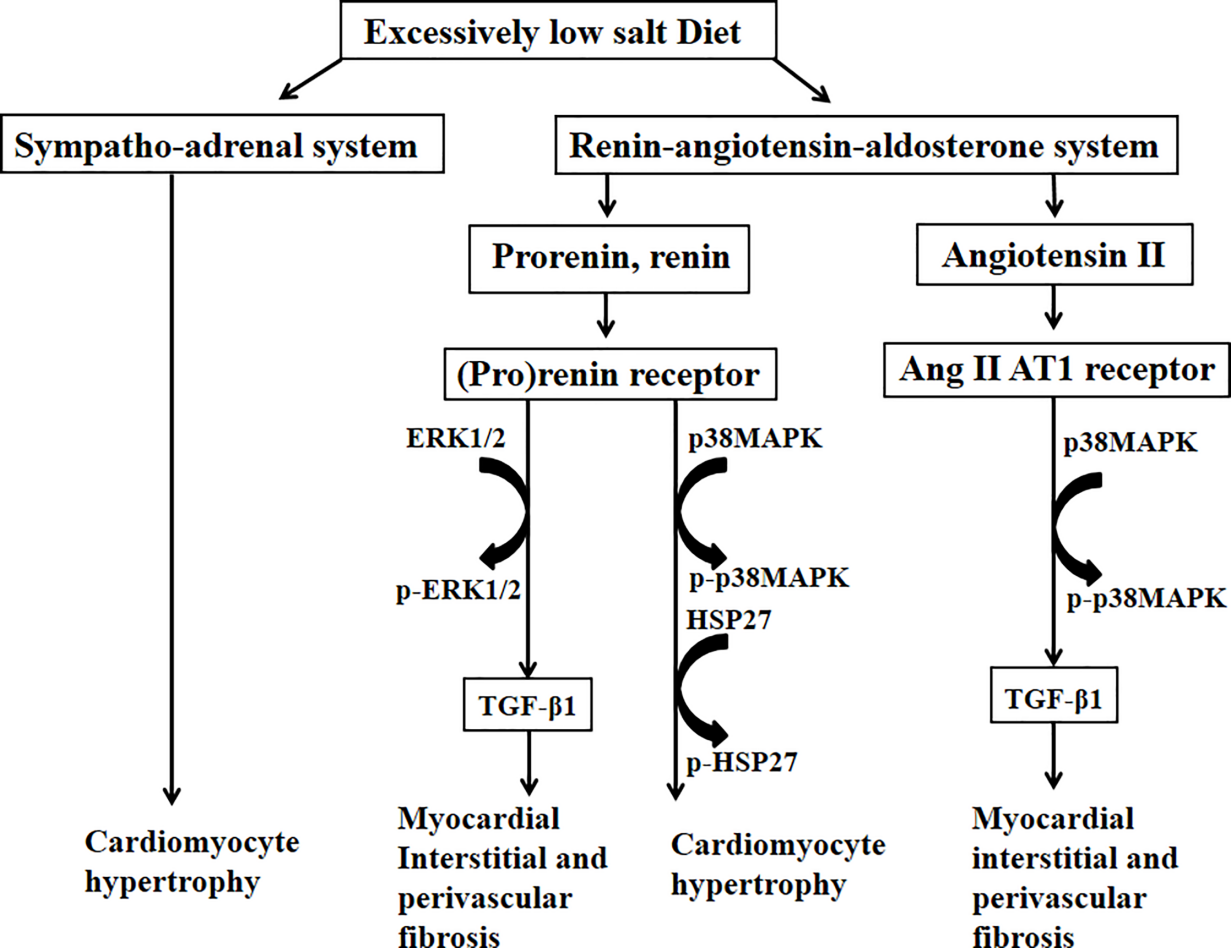
Proposed mechanism of excessively low salt diet-induced cardiac damage.

Fig 8 shows the proposed mechanism by which the excessively low salt diet leads to myocardial fibrosis, myocardial perivascular fibrosis, and cardiomyocyte hypertrophy, as noted in the present study. Signal transduction through (pro)renin receptor and angiotensin II AT1 receptors contribute to the effects of the saltless diet.

## Conclusions

In conclusion, the excessively low salt diet did not decrease the systolic blood pressure and increased the heart rate and activated the plasma renin-angiotensin-aldosterone and sympatho-adrenal systems as well as cardiac tissue prorenin-renin-(P)RR system. An excessively low salt diet is harmful to the heart and adequate salt restriction is required for the treatment of hypertension.
